# Heat loss augmented by extracorporeal circulation is associated with overcooling in cardiac arrest survivors who underwent targeted temperature management

**DOI:** 10.1038/s41598-022-10196-x

**Published:** 2022-04-13

**Authors:** Dong Hun Lee, Byung Kook Lee, Yong Soo Cho, Dong Ki Kim, Seok Jin Ryu, Jin Hong Min, Jung Soo Park, Kyung Woon Jeung, Hwa Jin Kim, Chun Song Youn

**Affiliations:** 1 Department of Emergency Medicine, Chonnam National University Hospital, Gwangju, Republic of Korea; 2 Department of Emergency Medicine, Chonnam National University Medical School, 160 Baekseo-ro, Dong-gu, Gwangju, 61469 Republic of Korea; 3 Department of Emergency Medicine, College of Medicine, Chungnam National University, Daejoen, Republic of Korea; 4 Department of Emergency Medicine, College of Medicine, The Catholic University of Korea, Seoul, Republic of Korea

**Keywords:** Cardiology, Neurology

## Abstract

We investigated the association of extracorporeal circuit-based devices with temperature management and neurological outcome in out-of-hospital cardiac arrest survivors who underwent targeted temperature management. Patients with extracorporeal membrane oxygenation and/or continuous renal replacement therapy were classified as the extracorporeal group. We calculated the cooling rate during the induction period and time-weighted core temperatures (TWCT) during the maintenance period. We defined the sum of TWCT above or below 33 °C as positive and negative TWCT, respectively, and the sum of TWCT above 33.5 °C or below 32.5 °C as undercooling or overcooling, respectively. The primary outcome was the negative TWCT. The secondary outcomes were positive TWCT, cooling rate, undercooling, overcooling, and poor neurological outcomes, defined as Cerebral Performance Category 3–5. Among 235 patients, 150 (63.8%) had poor neurological outcomes and 52 (22.1%) were assigned to the extracorporeal group. The extracorporeal group (β, 0.307; *p* < 0.001) had increased negative TWCT, rapid cooling rate (1.77 °C/h [1.22–4.20] vs. 1.24 °C/h [0.77–1.79]; *p* = 0.005), lower positive TWCT (33.4 °C∙min [24.9–46.2] vs. 54.6 °C∙min [29.9–87.0]), and higher overcooling (5.01 °C min [0.00–10.08] vs. 0.33 °C min [0.00–3.78]). However, the neurological outcome was not associated with the use of extracorporeal devices (odds ratio, 1.675; 95% confidence interval, 0.685–4.094).

## Introduction

Targeted temperature management (TTM) is a common approach to treat patients with comatose cardiac arrest after return of spontaneous circulation (ROSC)^1^. Various devices have been developed to achieve and maintain the target temperature within a certain range^2^. TTM devices work based on a temperature feedback system to avoid over or undercooling. Although guidelines recommend a target temperature of 33–36 °C^1^, the temperature may range within 0.5–1 °C above or below the target temperature^3–7^. Strict control of the target temperature at the selected range is essential for high-quality TTM^8^.

The use of extracorporeal circuit-based salvage therapy is increasing in patients that require critical care^9,10^. Continuous renal replacement therapy (CRRT) is commonly used in critically ill patients with acute kidney injury. Extracorporeal membrane oxygenation (ECMO) can be used as salvage therapy for patients with cardiopulmonary failure with reversible causes. CRRT and ECMO circuits are commonly sensitive to ambient heat loss. Although heat exchangers can be applied to the ECMO circuit and a CRRT system with a blood warmer has been developed, the conventional ECMO circuit has no temperature control function, and hypothermia still occurs during CRRT^11,12^. Therefore, CRRT and ECMO can be helpful to achieve the lower target temperature earlier in cardiac arrest survivors after ROSC. However, ambient heat loss from the circuit of CRRT and ECMO can affect maintenance of the target temperature during TTM. Although ECMO and CRRT for cardiac arrest survivors are used more frequently^13–15^, the effect of the interaction between ambient heat loss from the ECMO and/or CRRT circuits and automated temperature management devices on TTM has not been thoroughly evaluated.

To address this point, we recorded the core body temperature every minute during TTM. We hypothesized that TTM quality regarding temperature variability, such as overcooling and undercooling, was different between patients with CRRT and/or ECMO. We investigated the association between patients with an extracorporeal device and temperature characteristics, and between patients with an extracorporeal circuit device and neurological outcomes.

## Results

### Patient characteristics

Of the 308 out-of-hospital cardiac arrest (OHCA) survivors who underwent TTM, 5 patients died or were transferred during TTM, 11 patients underwent TTM with a target temperature other than 33 °C, 19 patients underwent TTM using a device other than Arctic Sun® (Energy Transfer Pads™; Medivance Corp, Louisville, CO, USA), and 38 patients had missing temperature data. Finally, 235 patients were included in the analysis.

Table 1 shows the baseline, cardiac arrest, post-ROSC, and initiation of TTM characteristics in the extracorporeal and no-extracorporeal groups. The extracorporeal group consisted of 52 patients; 44 patients with CRRT; 3 patients with ECMO; and 5 patients with both CRRT and ECMO. The extracorporeal group was more likely to have preexisting illness (hypertension, diabetes, and renal impairment), less initial shockable rhythm (10/52 vs. 81/183), higher epinephrine levels (3 mg [1–5] vs. 2 mg [0–4]), lower Glasgow Coma Scale (GCS) scores (3 [3–3] vs. 3 [3–5]), and higher sequential organ failure assessment (SOFA) scores (13 [12–15] vs. 10 [9–12]) than the no-extracorporeal group. Poor neurological outcomes differed between the extracorporeal and no-extracorporeal groups (40/52 vs. 110/183, *p* = 0.039). The extracorporeal group showed lower body temperature at the beginning of TTM (35.0 °C [33.7–36.0] vs. 36.3 °C [35.2–37.0]) and required shorter time to reach the target temperature (75 min [45–135] vs. 150 min [90–225]) (Table 1). Twenty-four patients in the extracorporeal group, who started extracorporeal support prior to TTM induction, showed more rapid cooling rate than patients in the no-extracorporeal group (Table 1).

**Table 1:** Comparison of baseline characteristics according to the use of extracorporeal devices.

Variables	Total (N = 235)	No-extracorporeal (N = 183)	Extracorporeal (N = 52)	*p*
Age, years	60.0 (50.0–70.0)	59.0 (48.0–69.0)	64.0 (54.0–73.8)	0.050
Male, n (%)	175 (74.5)	140 (76.5)	35 (67.3)	0.245
BMI, kg/m^2^	23.5 (21.0–26.1)	23.5 (21.3–26.0)	23.0 (20.8–26.2)	0.917
CRRT, n (%)	49 (20.9)	0 (0.0)	49 (94.2)	< 0.001
ECMO, n (%)	8 (3.4)	0 (0.0)	8 (15.4)	< 0.001
Preexisting illness, n (%)				
Coronary artery disease	40 (17.0)	28 (15.3)	12 (23.1)	0.268
Congestive heart failure	7 (3.0)	7 (3.8)	0 (0.0)	0.353
Hypertension	105 (44.7)	69 (37.7)	36 (69.2)	< 0.001
Diabetes	74 (31.5)	48 (26.2)	26 (50.0)	0.002
Pulmonary disease	14 (6.0)	13 (7.1)	1 (1.9)	0.315
Renal impairment	23 (9.8)	3 (1.6)	20 (38.5)	< 0.001
Cerebrovascular accident	23 (9.8)	15 (8.2)	8 (15.4)	0.202
Malignancy	14 (6.0)	12 (6.6)	2 (3.8)	0.741
Cardiac arrest characteristics				
Witness of collapse, n (%)	150 (63.8)	118 (64.5)	32 (61.5)	0.821
Bystander CPR, n (%)	154 (65.5)	126 (68.9)	28 (53.8)	0.065
Shockable rhythm, n (%)	91 (38.7)	81 (44.3)	10 (19.2)	0.002
Cardiac etiology, n (%)	165 (70.2)	127 (69.4)	38 (73.1)	0.734
Epinephrine, mg	2 (0–4)	2 (0–3)	3 (1–5)	0.001
Time to ROSC, min	27.0 (17.0–44.0)	26.0 (17.0–42.0)	31.5 (18.0–58.5)	0.237
Clinical characteristics after ROSC				
Glasgow Coma Scale	3 (3–5)	3 (3–5)	3 (3–3)	0.020
Lactate, mmol/L	8.6 (5.6–12.0)	8.1 (5.4–11.6)	10.5 (6.2–13.8)	0.072
Glucose, mg/dL	260.0 (189.0–337.0)	259.0 (191.0–326.0)	284.5 (171.0–386.0)	0.267
PaO_2_, mmHg	134.0 (86.6–223.6)	138.0 (90.0–223.6)	117.1 (75.3–235.3)	0.200
PaCO_2_, mmHg	44.0 (33.0–58.0)	44.0 (33.0–60.0)	44.5 (34.5–56.7)	0.708
SOFA score	11 (9–13)	10 (9–12)	13 (12–15)	< 0.001
Induction of TTM				
ROSC to TTM, min	271 (216–352)	271 (216–353)	281 (215–350)	0.672
CT at start TTM, ℃	36.0 (34.8–36.9)	36.3 (35.2–37.0)	35.0 (33.7–36.0)	< 0.001
Time to target temperature, min	135 (75–210)	150 (90–225)	75 (45–135)	< 0.001
Cooling rate, ℃/h	1.32 (0.81–2.00), 205*	1.24 (0.77–1.79), 181*	1.77 (1.22–4.20), 24*	0.005

Categorical variables were expressed as frequencies with percentages and continuous variables were expressed as medians with interquartile ranges.

*BMI* body mass index, *CRRT* continuous renal replacement therapy, *ECMO* extracorporeal membrane oxygenation, *CPR* cardiopulmonary resuscitation, *ROSC* return of spontaneous circulation, *SOFA* sequential organ failure assessment, *TTM* targeted temperature management, *CT* core temperature.

*included number for analysis.

#### Association between the time-weighted core temperature and use of an extracorporeal device

Table 2 shows the time-weighted core temperatures (TWCT) in the no-extracorporeal and extracorporeal groups during the maintenance phase of TTM. The extracorporeal group had lower positive TWCT (33.4 °C min [24.9–46.2] vs. 54.6 °C min [29.9–87.0]), higher negative TWCT (117.0 °C min [95.8–152.7] vs. 90.3 °C min [70.4–116.0]), and higher overcooling TWCT (5.01 °C min [0.00–10.08] vs. 0.33 °C min [0.00–3.78]) than the no-extracorporeal group (Table 2). The use of an extracorporeal device (β, 0.307; *p* < 0.001) was associated with increased negative TWCT in the whole groups and in the subgroup of CRRT (β, 0.270; *p* < 0.001), and ECMO groups (β, 0.444; *p* < 0.001) after adjusting for hypertension, SOFA score, and core temperature (CT) at the start of TTM.

**Table 2:** Time-weighted core temperatures in the extracorporeal groups.

Variables	Total (N = 235)	No-extracorporeal (N = 183)	Extracorporeal (N = 52)	*p*
Negative TWCT, ℃∙min	95.9 (74.7–128.2)	90.3 (70.4–116.0)	117.0 (95.8–152.7)	< 0.001
Positive TWCT, ℃∙min	49.2 (27.8–81.0)	54.6 (29.9–87.0)	33.4 (24.9–46.2)	0.003
Undercooling TWCT, ℃∙min	0.00 (0.00–0.00)	0.00 (0.00–0.00)	0.00 (0.00–0.00)	0.642
Overcooling TWCT, ℃∙min	0.47 (0.00–5.80)	0.33 (0.00–3.78)	5.01 (0.00–10.08)	0.002

Values are expressed as medians with the interquartile range.

*TWCT* time-weighted core temperature.

#### Association between extracorporeal devices and neurological outcomes

Table 3 shows the general characteristics stratified by neurological outcomes. The poor neurological outcome group was older (64.0 years [54.0–73.0] vs. 55.0 years [44.5–65.0]) with lower body mass index (22.9 kg/m^2^ [20.7–25.8] vs. 24.2 kg/m^2^ [22.1–26.4]) and more pre-existing illness (hypertension, diabetes, and pulmonary disease). This group was less witnessed (82/150 vs. 68/85), less likely to have bystander cardiopulmonary resuscitation (CPR) (91/150 vs. 63/85), shockable rhythm (28/150 vs. 63/85), and cardiac etiology (85/150 vs. 80/85). Patients in the poor neurological outcome group had longer time to ROSC (37.0 min [22.0–48.3] vs. 19.0 min [14.0–26.0]) and required higher epinephrine doses (3 mg [2–5] vs. 0 mg [0–2]). They had lower GCS scores (3 [3–3] vs. 5 [3–6]) and higher lactate (9.7 mmol/L [6.4–12.6] vs. 6.8 mmol/L [4.0–9.4]) and PaCO_2_ (50.5 mmHg [35.1–64.0] vs. 38.0 mmHg [31.6–46.5]) levels, higher SOFA scores (12 [10–13] vs. 10 [7–11]), shorter time from ROSC to TTM (258 min [207–324] vs. 294 min [226–366]), lower core temperature at start of TTM (35.5 °C [34.5–36.5] vs. 36.7 °C [36.0–37.3]), shorter time to target temperature (103 min [60–165] vs. 195 min [133–287]), and rapid cooling rate (1.5 °C/h [1.07–2.33] vs. 0.93 °C/h [0.61–1.39]).

**Table 3:** Comparison of baseline characteristics according to the neurological outcomes at 6 months.

Variables	Good (N = 85)	Poor (N = 150)	*p*
Age, years	55.0 (44.5–65.0)	64.0 (54.0–73.0)	< 0.001
Male, n (%)	68 (80.0)	107 (71.3)	0.143
BMI, kg/m^2^	24.2 (22.1–26.4)	22.9 (20.7–25.8)	0.011
CRRT, n (%)	10 (11.8)	39 (26.0)	0.010
ECMO, n (%)	4 (4.7)	4 (2.7)	0.407
Preexisting illness, n (%)			
Coronary artery disease	15 (17.6)	25 (16.7)	0.848
Congestive heart failure	3 (3.5)	4 (2.7)	0.706
Hypertension	28 (32.9)	77 (51.3)	0.006
Diabetes	14 (16.5)	60 (40.0)	< 0.001
Pulmonary disease	1 (1.2)	13 (8.7)	0.020
Renal impairment	6 (7.1)	17 (11.3)	0.289
Cerebrovascular accident	7 (8.2)	16 (10.7)	0.547
Malignancy	5 (5.9)	9 (6.0)	0.971
Cardiac arrest characteristics			
Witness of collapse, n (%)	68 (80.0)	82 (54.7)	< 0.001
Bystander CPR, n (%)	63 (74.1)	91 (60.7)	0.037
Shockable rhythm, n (%)	63 (74.1)	28 (18.7)	< 0.001
Cardiac etiology, n (%)	80 (94.1)	85 (56.7)	< 0.001
Epinephrine, mg	0 (0–2)	3 (2–5)	< 0.001
Time to ROSC, min	19.0 (14.0–26.0)	37.0 (22.0–48.3)	< 0.001
Clinical characteristics after ROSC			
Glasgow Coma Scale	5 (3–6)	3 (3–3)	< 0.001
Lactate, mmol/L	6.8 (4.0–9.4)	9.7 (6.4–12.6)	< 0.001
Glucose, mg/dL	251 (172–309)	267 (204–360)	0.061
PaO_2_, mmHg	126 (84–201)	136 (90–229)	0.388
PaCO_2_, mmHg	38.0 (31.6–46.5)	50.5 (35.1–64.0)	< 0.001
SOFA score	10 (7–11)	12 (10–13)	< 0.001
Induction of TTM			
ROSC to TTM, min	294 (226–366)	258 (207–324)	0.024
CT at start TTM, ℃	36.7 (36.0–37.3)	35.5 (34.5–36.5)	< 0.001
Time to target temperature, min	195 (133–287)	103 (60–165)	< 0.001
Cooling rate, ℃/h	0.93 (0.61–1.39), 78*	1.5 (1.07–2.33), 127*	< 0.001

Categorical variables were expressed as frequencies with percentages and continuous variables were expressed as medians with interquartile ranges.

*BMI* body mass index, *CRRT* continuous renal replacement therapy, *ECMO* extracorporeal membrane oxygenation, *CPR* cardiopulmonary resuscitation, *ROSC* return of spontaneous circulation, *SOFA* sequential organ failure assessment, *TTM* targeted temperature management, *CT* core temperature.

*included a number for analysis.

Multivariate logistic regression analysis revealed that the use of extracorporeal devices (compared with no extracorporeal device use) was not associated with neurological outcomes after adjusting for the etiology of cardiac arrest, witness of collapse, and GCS score. Compared with no extracorporeal device use, the use of CRRT or ECMO was not associated with neurological outcomes (Table 4).

**Table 4:** The association between poor neurological outcomes and the use of extracorporeal devices.

Variables	Odds ratio (95% confidence interval)	*p*
No-extracorporeal	Reference	
Extracorporeal	1.675 (0.685–4.094)	0.258
No-extracorporeal	Reference	
CRRT	2.171 (0.787–5.986)	0.134
ECMO or ECMO with CRRT	0.612 (0.110–3.401)	0.575

Adjusted with cardiac etiology, witness of collapse, and Glasgow Coma Scale score. *CRRT* continuous renal replacement therapy, *ECMO* extracorporeal membrane oxygenation.

## Discussion

The extracorporeal group had higher negative TWCT, rapid cooling rate, lower positive TWCT, and higher overcooling TWCT. The use of an extracorporeal device was independently associated with higher negative TWCT in the whole group and both subgroups of CRRT and ECMO. However, the use of extracorporeal devices showed no independent association with neurological outcomes.

Hypothermia during CRRT is a common adverse event and is presumed to be an independent factor for in-hospital mortality in critically ill patients^12,16,17^. However, hypothermia during CRRT may contribute to rapid induction of TTM. The shorter time required to reach the target temperature in the extracorporeal group is reasonable because this group had a lower body temperature at the beginning of TTM and demonstrated a rapid cooling rate during induction in the present study. In terms of achieving high-quality TTM, the extracorporeal circuit helps patients to reach the target temperature early. Although guidelines recommend performing TTM as soon as possible after ROSC, clinical trials have not proven a neuroprotective effect of early achievement of target temperature in cardiac arrest patients^1,18–21^. Moreover, it is uncertain whether early achievement of the target temperature with the contribution of the extracorporeal circuit is associated with neurological outcomes.

The extracorporeal group can be regarded to include patients who used a dual device for hypothermia. However, CRRT or ECMO has little or no functional capacity to control the body temperature; therefore, in contrast to the extracorporeal device, which was helpful during the induction period, CRRT or ECMO impaired proper temperature management during the maintenance period. The ambient heat loss from the circuit seemed to be more prominent in the ECMO subgroup rather than the CRRT subgroup because the circulating blood volume is larger, and the blood flow is faster in the ECMO group. The goal of a maintenance period is to maintain the core temperature at the target temperature. Strict temperature control, rather than the depth of the target temperature, has been suggested as a factor that defines high-quality TTM^8,22^. However, the extracorporeal group had a more biased core temperature pattern below the target temperature of 33 °C than the no-extracorporeal group.

During TTM, the core temperature is not always maintained within the target temperature range. In the present study, the positive TWCT directed above the target temperature were almost half of the negative TWCT directed below the target temperature. Positive TWCT should be minimized to achieve high-quality TTM because the increase in core temperature is associated with a potential risk for worse outcome. Although the extracorporeal group also showed positive TWCT, the significant difference in positive TWCT between the extracorporeal groups implies that the use of extracorporeal devices might effectively cancel heat production, indicating a positive aspect of extracorporeal devices. However, the overcooling in the extracorporeal group was higher than that in the no-extracorporeal group, whereas the undercooling was close to 0 in both groups. Although expected adverse events due to induced hypothermia in cardiac arrest survivors are uncommon^23,24^, the extracorporeal group requires monitoring to minimize overcooling.

As the extracorporeal group included patients who received CRRT and ECMO, patients in this group had a higher incidence of organ failure. Compared with the no-extracorporeal group, the extracorporeal group had a significantly higher SOFA score that corresponded to organ failure. Therefore, the extracorporeal group is expected to have poor neurological outcomes. However, the neurological outcomes were not independently associated with the use of extracorporeal devices. A recent clinical trial that showed no difference in neurological outcomes between patients with targeted hypothermia at 33 °C and targeted normothermia below 37.7 °C also indicated no association between neurological outcomes and depth of the target temperature^24^. Since heat loss from the extracorporeal circuit further lowered the core temperature below the target temperature, the use of extracorporeal devices might not be associated with neurological outcomes.

In this study, we calculated the TWCT based on the temperature measured every minute, which reflected the temperature dynamics between the extracorporeal and no-extracorporeal groups during TTM. However, this study has some limitations. This retrospective observational study could only identify the possible associations between the factors studied and was unable to identify any cause-and-effect relationships. Despite our efforts, there may be confounding factors that were not included in the multivariate analyses as the characteristics of the extracorporeal and no-extracorporeal groups and the ECMO and CRRT subgroups are different. ECMO was provided as an extracorporeal CPR or circulatory support after ROSC; thus, the ECMO pump had been already switched on before the induction of TTM in all patients with ECMO. However, not all patients who underwent CRRT had CRRT before the induction of TTM. Nevertheless, CRRT was at least running during the maintenance period. Finally, approximately 12% (38/308) of the patients were excluded because of data loss, which may have led to bias.

In conclusion, the patients with extracorporeal circuits achieved the target temperature earlier than those without extracorporeal circuits, and the core temperature distribution during maintenance period was further skewed below the target temperature of 33 °C. However, neurological outcomes were not independently associated with using the extracorporeal device.

## Methods

### Study design and population

This was a retrospective analysis of prospectively collected data from adult cardiac arrest survivors who had undergone TTM at Chonnam National University Hospital, a university-affiliated hospital in Gwangju, Korea, between October 2015 and December 2020. This study was approved by the Institutional Review Board of Chonnam National University Hospital (CNUH-2015-164). Informed written consent was obtained from the patient and/or the patient’s legal representative. This study was conducted in accordance with the principles of the Declaration of Helsinki^25^.

We included cardiac arrest survivors aged over 18 years who had undergone TTM with an Arctic Sun® system. We excluded patients who did not complete TTM due to transfer or death, patients with a target temperature other than 33 °C, patients who had undergone TTM with devices other than the Arctic Sun® system, and patients with missing core or water temperature data.

We classified patients who had ECMO and/or CRRT during TTM as the extracorporeal group. ECMO was used for extracorporeal CPR or circulatory support after ROSC. OHCA patients who had experienced collapse with bystander CPR, initial shockable rhythm, and 30 min of resuscitation effort (including basic and advanced life support) were indicated for extracorporeal CPR. Circulatory support with ECMO after ROSC was only provided to patients expected to have good neurological outcomes. The attending physician decided whether to apply ECMO. CRRT was used for patients with Kidney Disease Improving Global Outcomes stage 2 or 3. We used ECMO without a thermoregulator and CRRT with a thermoregulator.

### TTM and temperature measurement

We provided post-cardiac arrest care, including TTM, to comatose cardiac arrest survivors in accordance with the guidelines^1^. The target temperature of 33 °C was achieved as soon as possible after ROSC and then maintained for 24 h. After completion of the maintenance phase, patients were rewarmed to 36.5 °C at a rate of 0.25 °C/h. We administered midazolam and remifentanil for analgo-sedation and neuromuscular blockade with continuous infusion during TTM, to enhance TTM efficiency and to reduce brain metabolism. Amplitude-integrated electroencephalography was used to monitor subclinical seizures and propofol was administered to control seizures.

The Arctic Sun® system functions based on the core temperature measured using an esophageal probe and core temperature and water temperature were recorded automatically every minute throughout TTM.

### Data collection and definition

We obtained the following demographic data, and cardiac arrest and post-cardiac arrest profiles: age, sex, body mass index, preexisting illness, witness of collapse, bystander CPR, the first monitored rhythm (shockable or non-shockable), etiology of cardiac arrest (cardiac or non-cardiac), epinephrine dose during CPR, time to ROSC, GCS after ROSC, serum lactate after ROSC, blood glucose after ROSC, PaO_2_ and PaCO_2_ after ROSC, SOFA within the first day of cardiac arrest^26^, ECMO, CRRT, time from ROSC to start of TTM, body temperature at the start of TTM, and time to achieve target temperature.

**Figure 1:**
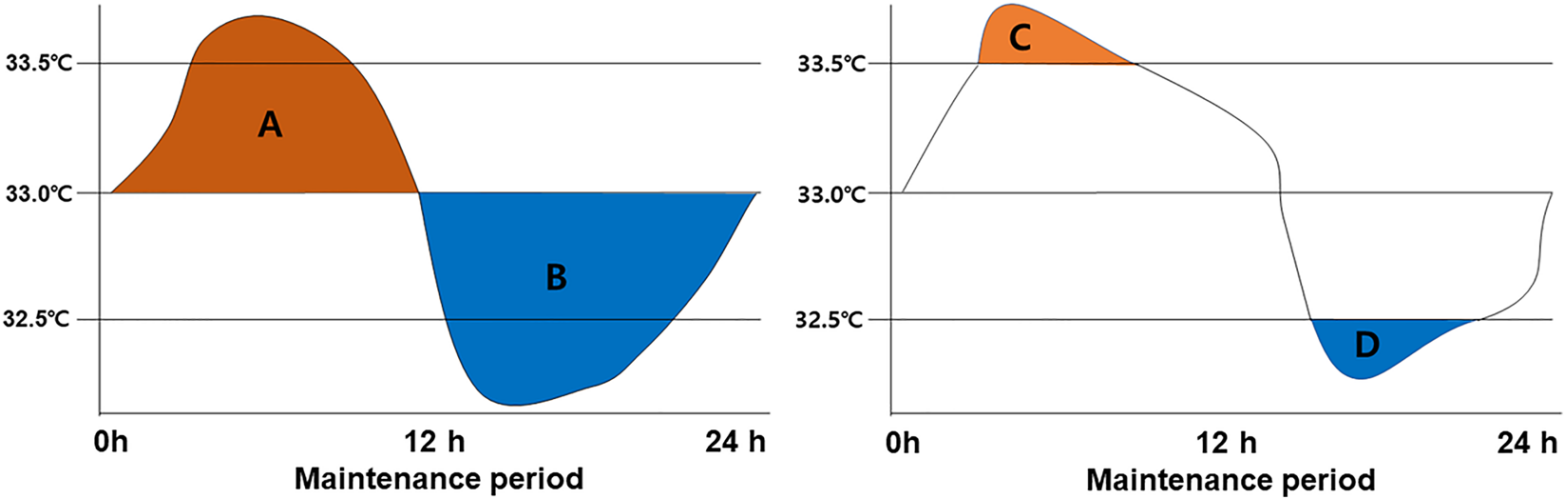
An example of time-weighted core temperature and core temperature variability during the 24 h of the maintenance period. The core temperature during the maintenance period was recorded every 1 min. Time-weighted core temperature was calculated to simultaneously quantify the degree of core temperature bias from the target temperature and exposure duration and is shown as a colored area. (**A**) Positive time-weighted core temperature. Degree and duration of core temperature above 33 °C. (**B**) Negative time-weighted core temperature. Degree and duration of core temperature below 33 °C. (**C**) Undercooling time-weighted core temperature over 33.5 °C. Degree and duration of core temperature above 33.5 °C. (**D**) Overcooling time-weighted core temperature under 32.5 °C. Degree and duration of core temperature below 32.5 °C.

The cooling rate during the induction period was calculated as the difference between the core temperature immediately before initiation of TTM and 33 °C to the time required to reach the target temperature. We calculated various indices to identify the temperature variability during the 24-h maintenance phase. We tried to simultaneously present the extent and time required for the core temperature to deviate from the target temperature of 33 °C during the maintenance phase. Therefore, we introduced the concept of TWCT, which can quantify core temperature and time. We calculated the TWCT based on the core temperature measured every 1 min (Fig. 1). Thereby, we defined the sum of TWCT > 33 °C as positive TWCT, whereas the sum of TWCT < 33 °C was defined as negative TWCT. We defined the sum of TWCT > 33.5 °C as the undercooling value, and the sum of TWCT < 32.5 °C as the overcooling value. The primary outcome was a negative TWCT.

We assessed the Cerebral Performance Category (CPC) scale at 6 months after cardiac arrest via phone interviews and recorded it as follows: CPC 1 (good performance), CPC 2 (moderate disability), CPC 3 (severe disability), CPC 4 (vegetative state), or CPC 5 (brain death or death)^27^. The secondary outcomes were poor neurological outcomes (CPC 3–5), cooling rate, positive TWCT, overcooling TWCT, and undercooling TWCT.

### Statistical analysis

Categorical variables, reported as frequencies with percentages, were compared between the two groups using χ^2^ tests with continuity correction in 2 × 2 tables or Fisher’s exact test, as appropriate. Continuous variables, reported as medians with interquartile ranges or mean with standard deviation according to the Shapiro–Wilk test, were compared between the two groups using Mann–Whitney *U* tests or independent t-tests, as appropriate.

To investigate the association between the use of extracorporeal devices and negative TWCT, we performed multivariate linear regression analyses. We performed multivariate logistic regression analyses using variables with *p* values < 0.2 (Table 1) and selected covariates for the linear regression analysis. We performed multivariate linear regression analyses in each subgroup of ECMO and CRRT in comparison with the no-extracorporeal group. Patients who received both ECMO and CRRT were included in the ECMO group.

We performed multivariate logistic regression analysis to investigate the association between the use of extracorporeal devices and neurological outcomes. We included all variables with *p* < 0.2 in the univariate analyses between neurological outcome groups (except cooling rate) as covariates in the logistic regression model. Enter method was used to develop the final adjusting model. The goodness of fit of the final model was evaluated using the Hosmer–Lemeshow test. We report logistic regression analysis results as odds ratios (OR) with 95% confidence intervals (CI). Data were analyzed using IBM SPSS Statistics 26.0 for Windows (IBM Corp., Armonk, NY, USA). A two-sided significance level of 0.05 was used to indicate statistical significance.

## Data Availability

Datasets generated and analyzed during the current study are available from the corresponding author on reasonable request.
